# Cytochrome *b* gene (*cytb*) sequence diversity in a *Microtus oeconomus* population from Bialowieza Primeval Forest

**DOI:** 10.1007/s13364-012-0096-7

**Published:** 2012-09-05

**Authors:** Michał J. Dąbrowski, Jan J. Pomorski, Joanna Gliwicz

**Affiliations:** Museum and Institute of Zoology, Polish Academy of Sciences, Wilcza 64, 00-679 Warsaw, Poland

**Keywords:** Bialowieza, Cytochrome *b* gene, Dispersal, Haplotype, Long-term study, Root vole

## Abstract

Based on published information about the glacial, postglacial, and recent distribution of the root vole, *Microtus oeconomus*, we hypothesized that a population inhabiting the pristine wetland in eastern Poland (Bialowieza Primeval Forest) might comprise a high diversity of haplotypes. The support for this hypothesis was provided by an analysis of partial *cytb* gene sequences from 149 voles sampled within a two-hectare plot during a nine-year study. In this population, we identified eight haplotypes (PLB1–PLB8), four of which were new to the root vole. These haplotypes were characterized by low nucleotide diversity (*π* = 0.0054, SE = 0.0019), the absence of transversional differences between sequences, and no changes in the encoded amino acid sequence: features suggesting a lack of immigration from the distant populations. The haplotype number and their frequency distribution in males and females did not differ significantly. An assessment of the persistence of matrilines in the local population throughout the study period revealed that the haplotype composition was relatively stable for only about 3 years. A more complete haplotype network for root voles in Europe was constructed by combining the newly identified haplotypes with the 45 previously described haplotypes. Two of the haplotypes detected in this study occupy key positions in this network: PLB5, as the closest link to the North European group, and PLB8, as an ancestor to many other Central European haplotypes.

## Introduction

According to the recent studies, several small mammal species, especially those of Arctic and temperate zone origin, persisted throughout the whole of the last glacial period in Central and Eastern Europe. The presence of the root vole *Microtus oeconomus* among these species is indicated by fossil data (Chaline 1987), phylogeographic evidence (Brunhoff et al. 2003), and distribution models of several rodent species of the northern temperate zone during the Last Glacial Maximum (LGM), based on climate simulation and niche distribution modeling (Fløjgaard et al. 2009). During this period, the distribution of both the root vole and lemmings extended as far north as Poland (see Brunhoff et al. 2003). Some sources even suggest that the root vole increased its range during the last glacial period (Chaline 1987; Kordos 1990). It seems that during the LGM (21000–17000 bp), climatic conditions suitable for this species occurred in Eurasia, from the Atlantic coast and broadly eastward, across Central Europe and the Russian Plain (Fløjgaard et al. 2009). Also during the postglacial period, the European range of the root vole was much broader than today, especially after its northward expansion when the climate ameliorated (Brunhoff et al. 2003).

Between the postglacial period and recent times, the geographic range of the root vole has contracted drastically, leaving behind threatened, geographically isolated populations in Hungary/Slovakia and the Netherlands (van Apeldoorn 1999). The contemporary European western boundary with its continuous range runs along the Oder River valley at the German–Polish border, but optimal habitats which maintain high density root vole populations begin farther east, in the eastern part of Poland (Buchalczyk and Pucek 1968; Raczyński et al. 1986; Borowski 1998; Gliwicz and Jancewicz 2004). The southern border of the present-day range of this species runs across the south of Poland (Sałata-Piłacinska 1990).

In this study, we examined the variability of the partial cytochrome *b* gene (*cytb*) sequence in the local population of *M. oeconomus* inhabiting natural sedgeland in the river valley of Bialowieza Primeval Forest (52°4′ N, 23°5′ E). This population has been the subject of a long-term study on relatedness and ecological features of voles experiencing temporal fluctuations in density (Dąbrowski 2010; Pilot et al. 2010). It is highly probable that Poland, and especially its eastern part where our studied population exists, is inhabited by root voles with exceptional genetic diversity due to (1) temporal continuity (glacial, postglacial, and recent) of the historical settlement of this vole in Central and Eastern Europe, (2) spatial continuity of its range from this region towards the east and north, and (3) an abundance of natural and pristine wetland habitats in this area. Therefore, the aims of our study were the following:

1. To estimate the richness of haplotypes in the population, which was expected to be high due to the pristine nature of the site and its location within the European range of this species
2. To assess the diversity and distribution of haplotypes in males and females, which could reflect intersexual differences in philopatry and dispersal
3. To combine the haplotypes found in the studied population with those previously identified and improve our understanding of the phylogeography of *M. oeconomus* in Europe

## Materials and methods

The studied root vole population inhabited a 1-ha plot (2 ha since 2006) which is situated within an extensive area (20 km^2^) of open sedgeland belonging to Bialowieza National Park in eastern Poland. For 9 years (2000–2008), this population was monitored by the use of the catch–mark–release method, and tissue samples were collected from all individuals upon first capture (for a more detailed description, see Pilot et al. (2010)).

The total DNA was extracted from tissue samples using the Genomic Mini Kit (A&A Biotechnology, Gdynia, Poland). For the analysis of *cytb* sequence diversity, we selected unrelated males and females present in the subsequent years (*n* = 169), which were identified using 20 microsatellite markers (Pilot et al. 2010).

A partial fragment of the mitochondrial *cytb* gene was obtained by PCR using the extracted DNA as template with the primer pair MoF (5′ CAGCATTCTCATCAGTAGCC 3′) and MoR (5′ GGGAAAAATAAAGCCAAAAT 3′) that was originally designed to distinguish (in cases of doubt) the young of *M*. *oeconomus* from those of *Microtus agrestis*. PCR amplification was carried out using the following thermocycle: 94 °C for 3 min then 40 cycles of 94 °C for 30 s, 55 °C for 30 s and 72 °C for 1 min, and followed by a final elongation at 72 °C for 5 min. The sequencing of the amplified fragments used the same primers (MoF, MoR) with a BigDye^TM^ Terminator Cycle Sequencing Ready Reaction Kit (Applied Biosystems, Austin, TX, USA). The sequencing products were purified according to the recommended protocol and analyzed on an ABI3500xL automated capillary sequencer.

The DNA sequences were edited and aligned, and the alignments were corrected manually in MEGA5 (Tamura et al. 2011). The final alignment was 475 base pairs (bp) in length. Although this fragment comprises only 41 % of the whole *cytb* sequence (1,140 bp), we found it suitable for our purposes as it permitted the detection of almost 80 % (*n* = 35) of the 45 known European root vole haplotypes that were identified using a more complete *cytb* sequence. Twenty samples were excluded from further analysis because of the detection (confirmed by additional testing) of more than one mitochondrial genotype per sample (i.e., heteroplasmy). Therefore, 149 sequences (84 females and 65 males) were used for the analyses. The number of haplotypes was calculated using DnaSP v5 (Librado and Rozas 2009).

The rates of evolutionary divergence (number of base substitutions per nucleotide site) between haplotypes PLB1–PLB8 were evaluated using the uncorrected *p* distance implemented in MEGA5. Evolutionary divergence between all pairs of haplotypes was estimated to assess their similarity. To determine the statistical significance of the obtained differences between pairs of haplotypes, the ANOVA test with the option for pairwise comparisons in the R package (R Development Core Team 2011) was used.

When assessing for the temporal persistence of haplotypes in the studied population, we calculated the probability that two randomly chosen females (FF) share the same haplotype in eight time intervals ranging from 0 to 8 years. For each of the FF pairs (*n* = 3,486), the response variable was binary coded: 0–if haplotypes were different; 1–if haplotypes were the same. Using the binary logistic regression implemented in Statistical Package for the Social Sciences (SPSS) 13.0 software (SPSS 2004), we assessed the impact of the time interval on the response variable. To determine the significance of differences in the mean value of the response variable between two time interval classes, 0–3 years (FF pairs *n* = 2,150) and 4–8 years (FF pairs *n* = 1,336), we used the Bootstrap *t* test implemented in Rundom Pro 3.14 (Jadwiszczak 2009). For the sake of statistical comparison, these calculations were repeated for males (MM) who cannot genetically ensure long-term persistence of this maternally inherited mtDNA marker, but can temporarily import it into the local population.

The sequences of the haplotypes found in the local population were compared with the corresponding region of *cytb* of *M. oeconomus* haplotypes from other European sites deposited in GenBank (Table 1). A median-joining network of all known haplotypes was constructed using Network 4.6 (Bandelt et al. 1999). The number of transitions and transversions between haplotypes as well as the nucleotide diversity of the *cytb* sequences were calculated using MEGA5.

**Table 1:** Geographic information and the GenBank accession numbers of *M. oeconomus* mitochondrial *cytb* gene haplotypes in Europe. Data are divided into two phylogroups following Brunhoff et al. (2003)

Group	Country	Haplotype	Identical haplotype	GenBank accession number
Central European	Poland	PLB1^a^		JQ627159
		PLB2^a^		JQ627160
		PLB3^a^	Moe01	JQ627161, GU987116 (Fink et al. 2010)
		PLB4^a^		JQ627162
		PLB5^a^	Pol-5	JQ627163, AY220013 (Brunhoff et al. 2003)
		PLB6^a^	Hun-Slo; Pol-4	JQ627164, AY220014 (Brunhoff et al. 2003), AY220012 (Brunhoff et al. 2003)
		PLB7^a^		JQ627165
		PLB8^a^	Pol-3	JQ627166, AY220010(Brunhoff et al. 2003)
		Pol-1		AY220008 (Brunhoff et al. 2003)
		Pol-2		AY220009 (Brunhoff et al. 2003)
		Moe02		GU954319 (Fink et al. 2010)
	Sweden	Swe-4	Nor-9	AY220003 (Brunhoff et al. 2003), AY220005 (Brunhoff et al. 2003)
	Norway	Nor-8		AY220004 (Brunhoff et al. 2003)
	Lithuania	Lith-1		AY220011 (Brunhoff et al. 2003)
	Netherlands	Neth-1		AY220006 (Brunhoff et al. 2003)
		Neth-2		AY220007 (Brunhoff et al. 2003)
North European	Norway	Nor-1	Nor-2	AY219981 (Brunhoff et al. 2003), AY219982 (Brunhoff et al. 2003)
		Nor-3	Nor-18	AY219983 (Brunhoff et al. 2003), DQ452142 (Brunhoff et al. 2006)
		Nor-4		AY219984 (Brunhoff et al. 2003)
		Nor-5		AY219985 (Brunhoff et al. 2003)
		Nor-6		AY219987 (Brunhoff et al. 2003)
		Nor-7	Fin-6;Fin-Swe	AY219988 (Brunhoff et al. 2003), AY219997 (Brunhoff et al. 2003), AY219989 (Brunhoff et al. 2003)
		Nor-10		DQ452134 (Brunhoff et al. 2006)
		Nor-11		DQ452135 (Brunhoff et al. 2006)
		Nor-12		DQ452136 (Brunhoff et al. 2006)
		Nor-13	Nor-14	DQ452137 (Brunhoff et al. 2006), DQ452138 (Brunhoff et al. 2006)
		Nor-15	Nor-16; Nor-17	DQ452139 (Brunhoff et al. 2006), DQ452140 (Brunhoff et al. 2006), DQ452141 (Brunhoff et al. 2006)
	Finland	Fin-1		AY219986 (Brunhoff et al. 2003)
		Fin-2		AY219990 (Brunhoff et al. 2003)
		Fin-3		AY219991 (Brunhoff et al. 2003)
		Fin-4		AY219992 (Brunhoff et al. 2003)
		Fin-5		AY219993 (Brunhoff et al. 2003)
	Sweden	Swe-1		AY219994 (Brunhoff et al. 2003)
		Swe-2	Swe-3	AY219995 (Brunhoff et al. 2003), AY219996 (Brunhoff et al. 2003)
	Belarus	Bel-1		AY219998 (Brunhoff et al. 2003)
	Russia	Rus-1		AY219999 (Brunhoff et al. 2003)
		Rus-2		AY220000 (Brunhoff et al. 2003)
		Rus-3		AY220001 (Brunhoff et al. 2003)
		Rus-4		AY220002 (Brunhoff et al. 2003)

^a^Haplotypes described in this study

## Results

### Genetic characteristics of the detected *M. oeconomus* haplotypes

Eight *cytb* gene haplotypes were detected in the examined population and named PLB1–PLB8 (GenBank accessions JQ627159-JQ627166). Of these haplotypes, four (PLB1, PLB2, PLB4, and PLB7) were new to *M. oeconomus*, whereas the other four had previously been observed in Poland, either at another site in Bialowieza or at a location of 130–150 km from this ancient woodland (Brunhoff et al. 2003; Fink et al. 2010). Haplotypes PLB3, PLB5, PLB6, and PLB8 corresponded to Moe01, Pol-5, Pol-4/Hun-Slo and Pol-3, respectively (Table 1).

Among the eight local haplotypes, the average number of base substitutions per nucleotide position was *π* = 0.0054 (SE = 0.0019; range 0.0107–0.0021). All variations between the nucleotide sequences of the analyzed haplotypes were situated in the third position of the codons. All of these changes were synonymous, and the amino acid sequence of the encoded cytochrome *b* did not vary between haplotypes. The average number of synonymous substitutions per synonymous site was *π* = 0.0223 (SE = 0.0076; range 0.0087–0.0444). All nucleotide sequence changes in the analyzed local haplotypes were transitions.

The level of evolutionary divergence between all pairs of haplotypes was evaluated. Haplotype PLB2 had the lowest and PLB8 had the highest similarity to the other haplotypes reported in this study. The evolutionary divergence values were significantly different only between these two haplotypes (ANOVA pairwise test *t* = −4.08; *p* = 0.004).

### Persistence of the haplotypes in a local population

**Fig. 1:**
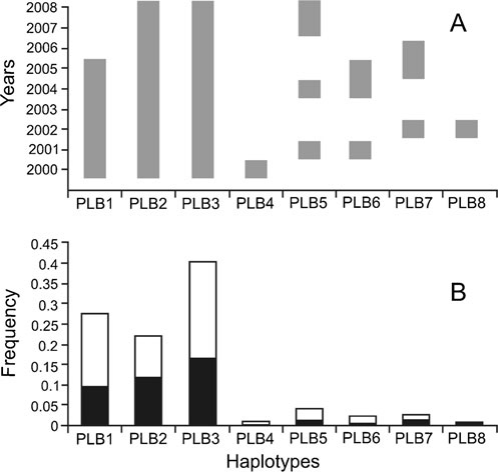
Temporal persistence (**a**) and frequency distribution (**b**) of eight mitochondrial cytochrome *b* gene haplotypes in the local root vole population in a plot situated on open sedgeland in Bialowieza Primeval Forest. *Black* males and *white* females

In the studied population, three haplotypes (PLB1–PLB3) were the most persistent: PLB2 and PLB3 were observed in all years of the study, and PLB1 in the first 6 years (2000–2005). Some haplotypes occurred in 1 year only (PLB4 and PLB8), while others were observed in 3–4 years (PLB5, PLB6, and PLB7), but not continuously (Fig. 1a).

The three most persistent haplotypes were also the most common in the population (PLB1–PLB3), occurring in 20–40 % of each voles, whereas the more ephemeral haplotypes were found in only 1–5 % of individuals (Fig. 1b). No statistically significant difference was found between the frequency distribution of haplotypes in males and females (*χ* ^2^ = 6.57; *df* = 7; and *p* = 0.47) most probably because of the even distribution of the three dominant haplotypes (PLB1–PLB3).

**Fig. 2:**
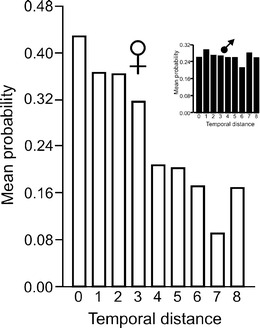
Mean probability of sharing the same haplotype by pairs of females separated by different temporal distances (in years), as assessed by binary logistic regression. Note the sharp decline in the probability in female pairs separated by more than 3 years. The distribution of the similarly calculated probability for pairs of males is shown as an insert for comparison

In order to assess the persistence of matrilines at the studied site, we compared haplotype similarity in the pairs of females separated by different temporal distances (0–8 years). If the same matrilines were present throughout the study period, the probability of sharing the same haplotype should not decrease with time. However, pairwise analysis revealed that the probability that a pair of females share the same haplotype did decrease significantly with increasing time interval (*B* = −0.866; SE = 0.037; Wald = 545.110; *df* = 1; and *p* < 0.001). The probability was relatively high in the first time interval class (0–3 years; mean = 0.366), but subsequently it decreased sharply and was significantly lower in the second time interval class (4–8 years; mean = 0.183) (Bootstrap *t* test *t* = 11.706; *p* < 0.001). If the males in the population inherited mtDNA only from the local females, the temporal distribution of the probability of pairs of MM sharing a haplotype should be similar to that of females. However, the probability for MM pairs remained stable throughout the analyzed period, indicating other sources of haplotype diversity in males (Fig. 2).

### Placing identified haplotypes in the European network

**Fig. 3:**
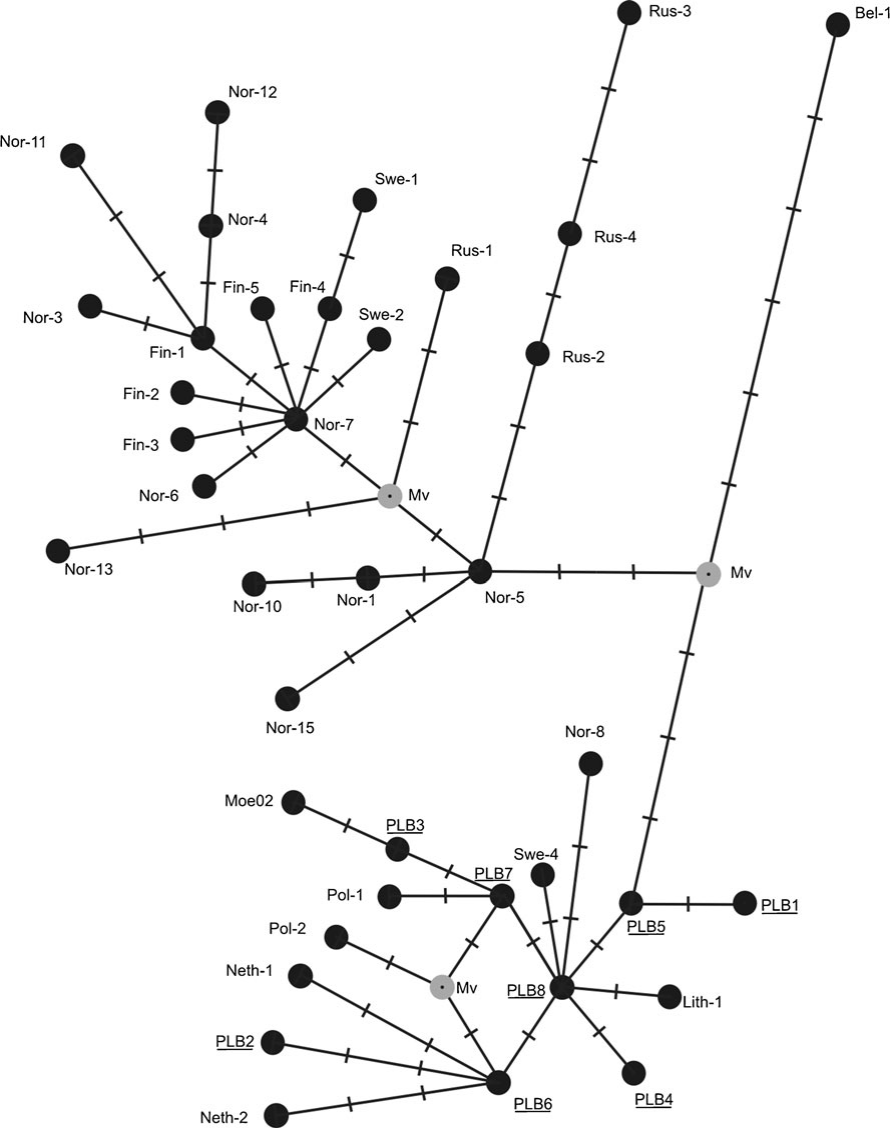
Median-joining network of 53 cytochrome *b* gene sequences (from this study and GenBank accessions) which, after the elimination of replicates, represents 39 haplotypes of *M. oeconomus* found in Europe. On the connecting *lines*, one division corresponds to a single nucleotide substitution. *Mv* median vector (hypothetical sequence)

A haplotype network for Europe based on *cytb* sequences from GenBank, i.e., those identified previously plus PLB1–PLB8, clearly showed two separate phylogroups: one composed of haplotypes from Central European sites and southern Scandinavia and the other comprising haplotypes from Russia and northeastern Fennoscandia (Fig. 3). These two groups are linked by the connection between PLB5 from Bialowieza (and another site in eastern Poland) and Nor-5 found in northern Norway.

PLB8, another haplotype from Bialowieza and the vicinity, gave rise to many other haplotypes of the Central European group: the local PLB4, PLB6 (detected also in Hungary and Slovakia), and PLB7, as well as those reported from Sweden (Swe-4), Norway (Nor-8), and Lithuania (Lith-1). PLB6, in turn, was the source of two haplotypes from the Netherlands (Neth-1 and Neth-2) and the local PLB2. Pol-1 and Pol-2, reported from western and northern Poland, respectively, and another haplotype previously identified in Bialowieza (Moe02) were not found in the studied population. In addition, the new haplotype network demonstrated that a haplotype from Belarus is (in the analyzed region of the *cytb* sequence) very different from all the others (Fig. 3).

## Discussion

Our expectations of the high diversity of root vole haplotypes in Bialowieza Primeval Forest, based on the glacial and postglacial history of the species range (Chaline 1987; Kordos 1990) and phylogeography (Brunhoff et al. 2003; Haring et al. 2011), were fully justified by the results of this study. Eight haplotypes (based on partial *cytb* gene sequences) detected within a small area (1–2 ha) constitute almost 20 % of all haplotypes found in Europe so far and in a considerable part of those known as Central Europe. Moreover, among these sequences, four haplotypes were new for root voles in Europe. Had the full sequence of *cytb* been analyzed, it is probable that the number of haplotypes detected in our study would have been even greater. It might be expected that the diversity of root vole haplotypes in Europe will increase as more sites are sampled, although it is difficult to estimate how much. In the case of two more thoroughly investigated *Microtus* species, the haplotype diversity and distribution is somewhat different: 116 haplotypes of the common vole (Tougard et al. 2008) and 66 of the field vole (Jaarola and Searle 2002) have been identified in Europe, but the ranges of these species extend more towards the south and west than that of the root vole. The uniqueness of the studied site is further confirmed by the fact that some of the eight reported haplotypes seem to be ancestral for other Central European cytochrome *b* gene sequences.

The local haplotype richness stems from the special geographical position of the plot within the contact zone between the Central and North European phylogroups of the root vole (Brunhoff et al. 2003). Phylogeographic studies of the two other temperate zone arvicolids, *M. agrestis* and *Myodes glareolus*, also point to the region of eastern Poland and Lithuania as the zone of contact between Central European and North or East European lineages (Jaarola and Searle 2002; Wójcik et al. 2010).

Another source of the high haplotype diversity in Bialowieza may be the large wetland on which the study plot was situated, which is an optimal habitat for root voles. This ancient habitat harbors an abundant *M. oeconomus* population and has probably done so since the postglacial period. The conservative genetic character of this population is indicated by the low nucleotide diversity (*π* = 0.0054, SE = 0.0019) observed between the eight haplotypes, with only transitional differences between the sequences that do not change the cytochrome *b* amino acid sequence. In addition, the minor differentiation between local haplotypes indicates a lack of gene flow from distant locations provided by long-distance immigrants. This may be explained by the relatively high isolation of the patches of wetland habitat suitable for the root vole. A dry habitat matrix may seriously impede long-distance migration by these hygrophilous voles (Tast 1966; Steen 1994).

The temporal persistence of some haplotypes in the studied population was short and discontinuous, which highlights the advantages of a longer sampling period than is usually practiced in phylogenetic studies for the registration of all haplotypes at individual sites. The short-lived occurrence of some haplotypes was almost certainly an effect of frequent density crashes (Gliwicz and Jancewicz 2004; Zub et al. 2012), followed by genetic bottlenecks reported for this population (Pilot et al. 2010). Evidently, the less common haplotypes disappeared from the plot after such episodes, but survived elsewhere in the sedgeland, and were later reintroduced to the local population by the dispersing female voles that were more likely to settle and reproduce at low density (Dąbrowski 2010). This scenario is supported by the high haplotype similarity in pairs of females separated by 0–3 years that quickly eroded afterwards. We interpret this as an effect of philopatry and nepotism of the female kin that promotes maintenance of extended families in a local microhabitat for at least several years until serious flooding or a population crash occurs. After such event, a different set of females might have settled in a local habitat.

In males, the composition of haplotypes in a local population may be formed in two ways: their inheritance from local mothers and the influx of male immigrants, which is common in many vole species (Greenwood 1980; Ishibashi and Saitoh 2008; Gauffre et al. 2009). The significant difference in long-term haplotype dynamics between females and males (as revealed by the temporal changes in the probability of pairs of individuals sharing a haplotype) indicates high mobility of males to and from the local plot.

Due to these well-established intersexual differences in vole mobility, we expected to observe a lower number of haplotypes carried by more sedentary females than by males in a local population, as found by Borkowska and Ratkiewicz (2008) in their study on common voles. The fact that we did not find such a difference may be explained in two ways. First, any difference in the total number of haplotypes detected in males and females in a short-term study may disappear if the study period is prolonged. Second, due to frequent flooding of their habitat (as mentioned above), the female root voles are probably more mobile than the female common voles that live in drier habitats (but perhaps not those inhabiting in frequently disturbed cultivated fields—see Gauffre et al. (2009)).

The haplotype network (median-joining) constructed here (Fig. 3) covers only two of the four phylogroups of *M. oeconomus* reported by Brunhoff et al. (2003). According to these authors, the division into Central European, North European, Central Asian, and Beringian phylogroups was initiated by events predating from the last glaciation. Haplotypes from Poland form one group with the other haplotypes from Central Europe. The other European phylogroup is formed by haplotypes found in North and North East Europe (Sweden, Norway, Finland, and Russia up to the Ural Mountains).

The haplotypes PLB5 and PLB8 seem to occupy key positions within the Central European haplotype network. The former is most closely related to the northern phylogroup which is evident in both the phylogenetic tree proposed by Brunhoff et al. (2003) (in their Pol-5) and in our new network (Fig. 3), whereas the latter has given rise to many other Central European haplotypes. Among the haplotypes identified in the present study, PLB8 showed the highest similarity to (and lowest divergence from) all the others. Moreover, this haplotype and its immediate derivatives, PLB6 and PLB7, were the source of haplotypes identified in the Netherlands, Lithuania, Hungary, Slovakia, southern Sweden, and southern Norway.

Interestingly, haplotype Bel-1 from Belarus, which according to Brunhoff et al. (2003) belongs to the North European group, might be quite distant from it. From our analysis of the partial *cytb* sequence, it seems that Bel-1 forms some kind of an outgroup. Finally, with regard to the probable ancestors of Pol-2, the close link between Pol-1 and Pol-2 proposed by Brunhoff et al. (2003) suggests that Pol-2 was derived from PLB7 rather than from PLB6.

It may be concluded that (1) in areas of high genetic diversity, longer sampling periods than those usually employed in phylogenetic studies permit the more complete registration of haplotypes present at individual sites; (2) analysis of the full *M. oeconomus cytb* sequence would probably identify more than eight haplotypes on our study site, although the high variability in the examined gene region in relation to that of the whole sequence suggests that the number of missed haplotypes is unlikely to be high; and (3) based on the haplotype diversity data from this and previous studies, the intensified sampling in the region of eastern Poland, Lithuania, Belarus and northwestern Russia, i.e., in the expected contact zone between three root vole phylogroups, is likely to reveal high haplotype richness.
